# Barbed suture vs conventional tenorrhaphy: biomechanical analysis in an animal model

**DOI:** 10.1007/s10195-014-0333-8

**Published:** 2015-01-28

**Authors:** A. Clemente, F. Bergamin, C. Surace, E. Lepore, N. Pugno

**Affiliations:** 1 Department of Hand, Plastic and Reconstructive Surgery, Maria Vittoria Hospital, Turin, Italy; 2 Laboratory of Bio-inspired Nanomechanics “Giuseppe Maria Pugno”, Department of Structural, Building and Geotechnical Engineering, Politecnico di Torino, Turin, Italy; 3 Laboratory of Bio-inspired and Graphene Nanomechanics, Department of Civil, Environmental and Mechanical Engineering, University of Trento, Via Mesiano 77, 38123 Trento, Italy; 4 Centre of Materials and Microsystems, Bruno Kessler Foundation, Via Santa Croce 77, 38122 Trento, Italy; 5 School of Engineering and Materials Science, Queen Mary University, Mile End Rd, London, E1 4NS UK

**Keywords:** Barbed suture, Breaking force, Tenorrhaphy, Biomechanical testing

## Abstract

**Background:**

The advantages of barbed suture for tendon repair could be to eliminate the need for a knot and to better distribute the load throughout the tendon so as to reduce the deformation at the repair site. The purpose of this study was to evaluate the breaking force and the repair site deformation of a new barbed tenorrhaphy technique in an animal model.

**Materials and methods:**

Sixty porcine flexor tendons were divided randomly into three groups and repaired with one of the following techniques: a new 4-strand barbed technique using 2/0 polypropylene Quill™ SRS or 2/0 polydioxanone Quill™ SRS and a modified Kessler technique using 3/0 prolene. All tendons underwent mechanical testing to assess the 2-mm gap formation force, the breaking force and the mode of failure. The percentage change in tendon cross-sectional area before and after repair was calculated.

**Results:**

The two-sample Student *t*-test demonstrated a significant increase in 2-mm gap formation force and in breaking force with barbed sutures, independently from suture material, when compared to traditional Kessler suture. Concerning the tendon profile, we registered less bunching at the repair site in the two barbed groups compared with the Kessler group.

**Conclusions:**

This study confirms the promising results achieved in previous ex vivo studies about the use of barbed suture in flexor tendon repair. In our animal model, tenorrhaphy with Quill™ SRS suture guarantees a breaking force of repair that exceeds the 40–50 N suggested as sufficient to initiate early active motion, and a smoother profile at the repair site.

*Level of evidence* Not applicable.

## Introduction

An ideal tendon repair would ensure a sufficient breaking force with a minimal deformity in the tendon repair site to allow early passive and active motion so as to reduce tendon adhesions and improve the functional outcome. In a conventional tenorrhaphy, knots are the weak point of tendon repair, being operator dependent and causing decreased tendon apposition. Increased suture diameter and number of knots increases the force of repair but also the tendon cross-sectional area, causing an increased gliding resistance. To avoid the potential weakness from knots, and to improve the interaction between tendon tissue and suture materials, it is proposed that barbed sutures could be utilized.

In 1967, McKenzie described the first account for the use of an internal multiple barbed suture to repair flexor tendons in a canine model [1, 2]. Recently, with the improvement in biomaterial and US Food and Drug Administration approval of barbed nylon, polydioxanone and polypropylene sutures, a renascent interest in this kind of suture material was registered. Quill™ Self-Retaining System (SRS) (Angiotech, Vancouver, BC, Canada) is a barbed bidirectional suture, created using absorbable and non-absorbable materials, with barbs spiraling around the central core suture and armed with a surgical needle on each end. The barbs anchor tissues so Quill™ SRS does not require knots to approximate opposing edges of a wound.

Up until now, few studies concerning the breaking force^1^ of tenorrhaphy with barbed sutures have been published, and all in cadaver or animal models. The purpose of this study was to evaluate the breaking force and repair site characteristics of a new 4-strand technique using Quill™ SRS, compared with the traditional modified Kessler technique in flexor tendon repair in a porcine model.

## Materials and methods

**Fig. 1:**
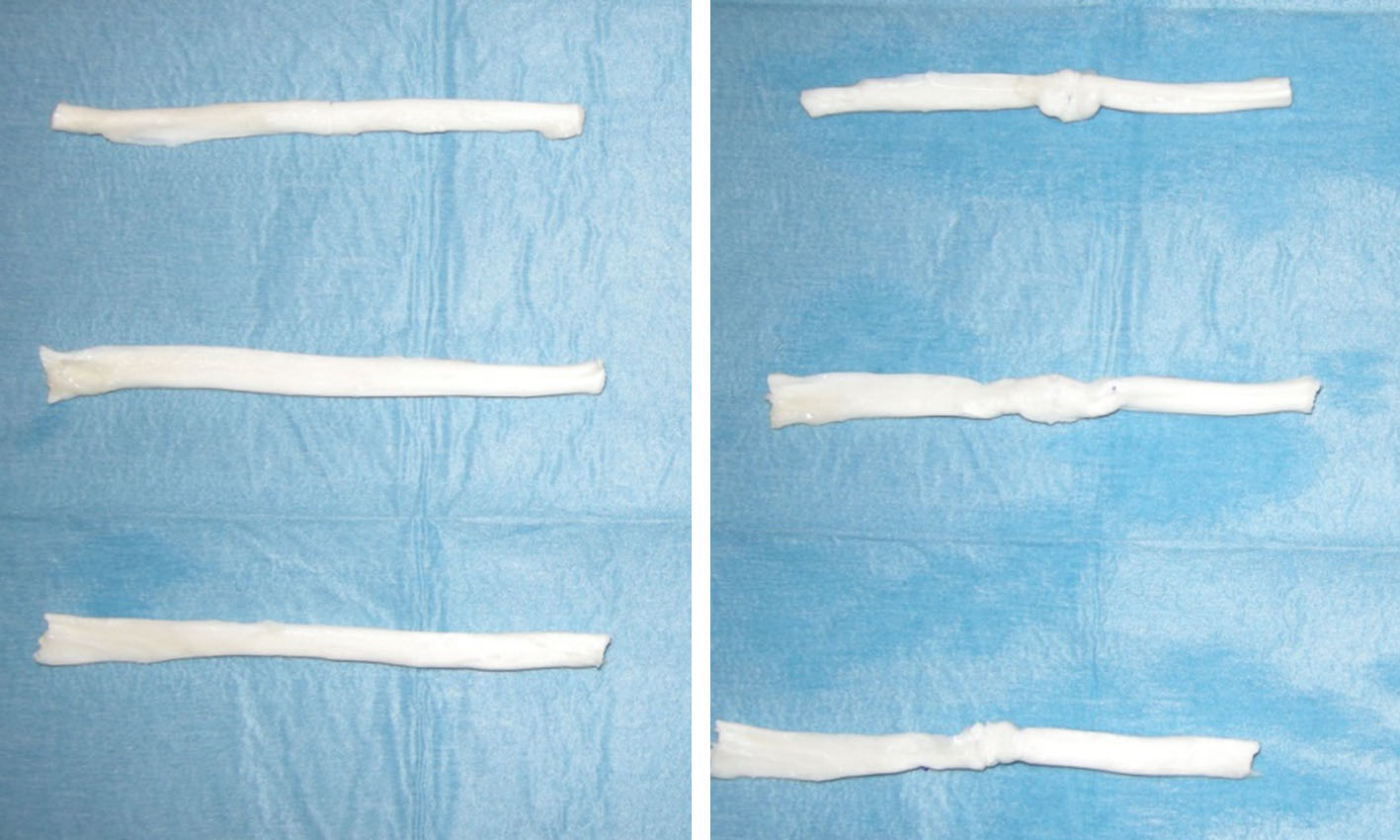
Tendons before and after the suture: repair site distortion with the modified Kessler technique (*above*), with the new 4-strand barbed technique with 2/0 polypropylene Quill^TM^ SRS (*center*) and with the new 4-strand barbed technique with 2/0 PDO Quill^TM^ SRS (*below*) in comparison with uninjured tendon (*on the left*)

Sixty tendons of similar size were obtained from the forelegs of adult pigs for slaughter. The pig model was chosen for the similarity in structure and strength to a human tendons [3]. Tendons were examined for abnormalities, such as synovitis and degeneration, and were rejected if an anomaly was present. Sheaths were excised and tendons were stored with refrigeration. During tendon harvest, preparation and repair (Fig. 1), desiccation was prevented with application of normal saline. Each tendon was transected at the midpoint and was measured by a single observer with a digital caliper to determine the pre-repair (*A*_PR_) and post-repair (*A*_R_) cross-sectional area. The cross-sectional area was calculated assuming an elliptic cross-sectional area, i.e., equal to π*ab*, where *a* and *b* are equal one-half tendon height and width, respectively. The change between the post-repair and the pre-repair cross-sectional areas was determined as (*A*_PR_−*A*_R_)/*A*_PR_ (%). A single surgeon harvested all tendons and performed all sutures.

**Fig. 2:**
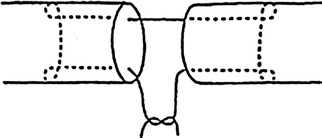
The modified Kessler technique used in group A

**Fig. 3:**
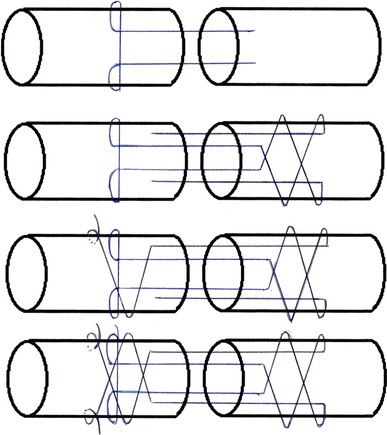
The new 4-strand barbed technique used in groups B and C

The tendons were randomly assigned to three repair groups: 20 tendons sutured using 3/0 prolene with a 2-strand modified Kessler technique (group A) (Fig. 2); 20 using 2/0 polypropylene Quill™ SRS with a new 4-strand barbed technique (group B) (Fig. 3); 20 using 2/0 polydioxanone (PDO) Quill™ SRS with the same new 4-strand barbed technique (group C). No suture was performed in the epitenon.

The 2/0 Quill™ SRS barbed suture was chosen because it has a breaking force that most closely resembles that of 3/0 unbarbed suture [4], according to the manufacturer’s data. After testing the new 4-strand barbed technique with 2/0 polypropylene Quill™ SRS, the same tenorrhaphy was performed with 2/0 PDO Quill™ SRS, a monofilament synthetic absorbable suture, to assess whether there was an improvement in breaking force with this suture material.

For knotless tendon repair, the following new technique was used (Fig. 3). The beginning is like a Kessler technique, but each needle enters the lateral wall of the proximal tendon stump perpendicular to the fibrils before turning 90 ° and exiting the stump. In the distal stump, each needle was advanced parallel to the direction of the fibrils for a distance of 0.5 cm before exiting the tendon surface. Next, each needle was used to make two transverse passes perpendicular to the direction of the tendon fibrils. Each needle was then reintroduced into the tendon and advanced parallel to the fibrils to traverse the injury site and enter the opposite end of the tendon for a distance of 0.5 cm before exiting the tendon surface. Again, two transverse passes were made to anchor the suture, and following the second pass, the excess suture and needle were cut off. This process resulted in a knotless repair with four strands crossing the injury site and four transverse passes at each end of the tenorrhaphy.

All biomechanical tensile tests were done in the Laboratory of Bio-inspired Nanomechanics “Giuseppe Maria Pugno” (Politecnico di Torino, Italy) with an air temperature of 22 ± 1 °C and 31 ± 2 % of relative humidity. Tendons were kept moist up until the test with normal saline.

**Fig. 4:**
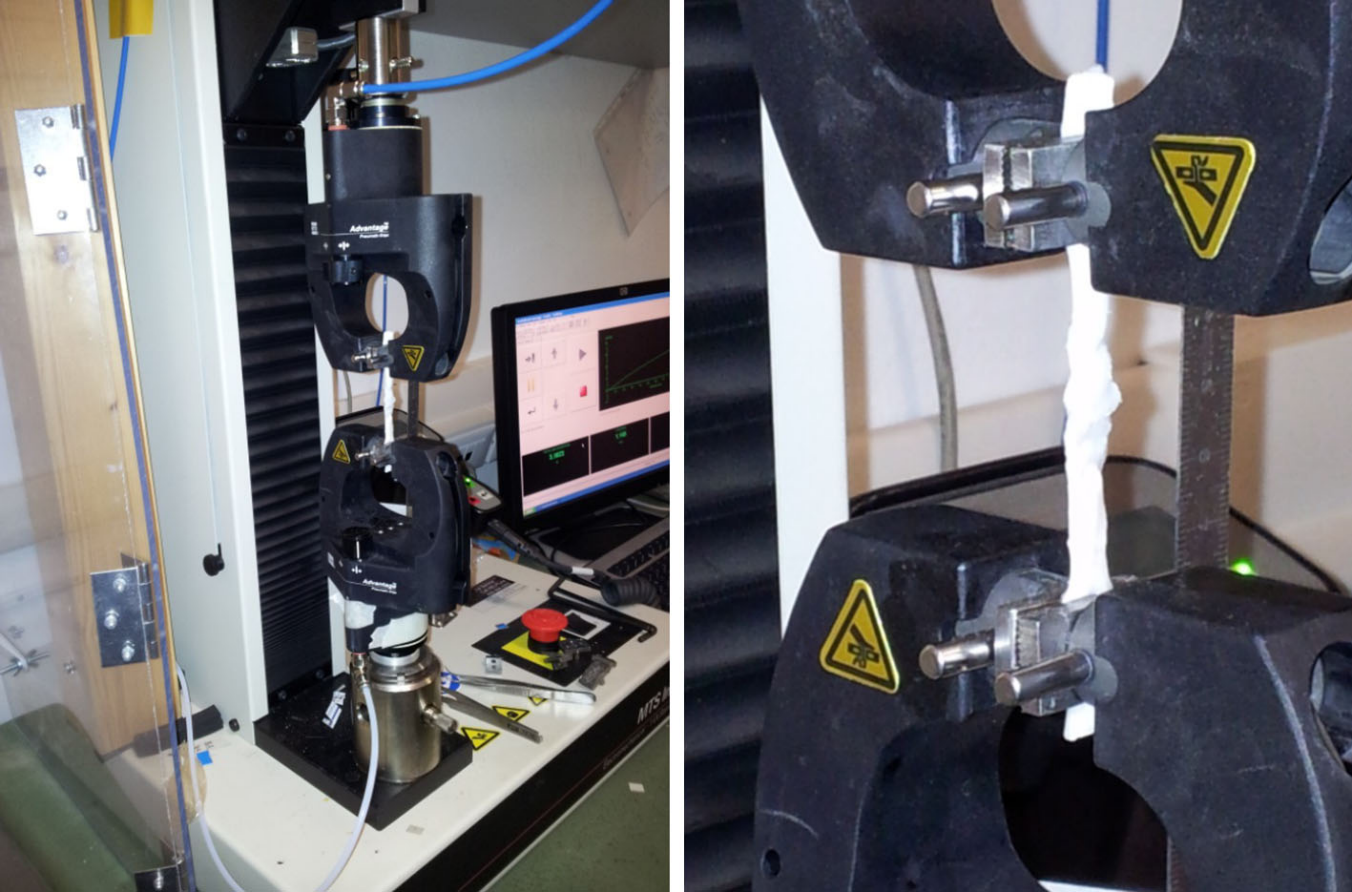
Flexor tendon in tension on MTS with pneumatic saw-tooth-shaped clamps holding the tendon

The tensile tests were conducted using a testing machine (Insight 1 kN, MTS, Minnesota, USA), equipped with a 100-N cell load with pneumatic saw-tooth-shaped clamps (closure pressure of 275.6 kPa), which prevent tendon slippage during testing (Fig. 4). The clamps were brought to zero tension before starting mounting tendons, which were placed between clamps defining an initial length *l*_0_ of 50 mm. Once tendons were in place, a preload of ~2 N was applied by slightly raising the actuator, leaving the tendons loose to properly extend between the clamps, without placing significant tension on the repair, in accordance with previously published papers [5, 6]. The specimens were pulled until they completely broke using a displacement-controlled uniaxial tension at a constant rate of 20 mm/min, as in previous studies [7]. This preload and rate were selected because they best simulate forces acting on an immobilized tendon during active flexion.

In addition, tensile tests were performed for the suture materials, fixing the same initial length *l*_0_ and the same constant rate of 20 mm/min, without the 2-N preload.

The computer program Test Works 4 (MTS, Minnesota, USA) recorded the experimental data of the applied tensile force and displacement. All tendons underwent mechanical testing to assess the 2-mm formation force, which was calculated using a bar scale placed near the repaired tendon and captured with a DCR SR55E SONY digital video camera. Linear traction continued until the suture materials were ruptured or tendons failed, and the breaking force was recorded immediately before failure.

A two-sample Student *t-*test was performed to determine whether there was a significant difference in load at 2-mm gap formation, maximum load or pre-repair areas among the three repair groups. Differences at the *P* ≤ 0.01 level were considered significant.

## Results

The force corresponding to 2-mm gap formation and to breaking of the suture, the mode of sample failure, the pre-repair (*A*_PR_) and post-repair (*A*_R_) cross-sectional areas and the changes (%) in tendon dimensions are listed in Table 1. Table 2 reports the mechanical data of the suture materials alone. All values are reported as mean ± SD.

**Table 1:** Results of biomechanical tensile tests of tendon repairs including tensile force of 2-mm gap formation, the breaking force, the mode of sample failure, the pre-repair (*A*_PR_) and post-repair (*A*_R_) cross-sectional area and the changes (%) in tendon dimensions

Repair technique	Tensile force (*N*)		Failure mode (observed number)		Repair site cross-sectional area (mm^2^)		
	2-mm gap formation	Breaking force	Suture breakage	Suture pull-out	Pre-repair (*A*_PR_)	Post-repair (*A*_R_)	Change (%)
Group A	21.2 ± 5.9	28.2 ± 6.2	12	8	12.4 ± 3.1	24.7 ± 7.6	99.6
Group B	38.2 ± 9.3	50.3 ± 9.9	20	0	14.6 ± 2.8	25.7 ± 10.0	76.3
Group C	41.0 ± 11.4	61.5 ± 11.0	20	0	15.4 ± 2.3	25.0 ± 6.1	61.8

Group A: modified Kessler technique. Group B: 4-strand barbed technique with 2/0 polypropylene Quill™ SRS. Group C: 4-strand barbed technique with 2/0 PDO Quill™ SRS

**Table 2:** Results of biomechanical tensile tests of suture materials alone

Suture material	Tensile force (*N*)
	Breaking force
3/0 prolene	23.5 ± 0.9
2/0 polypropylene Quill™ SRS	27.1 ± 1.2
2/0 PDO Quill™ SRS	28.3 ± 1.0

**Fig. 5:**
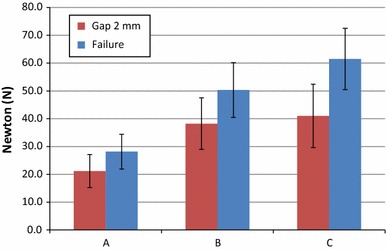
Comparison of forces among tendon repair techniques (*A* Kessler suture, *B* barbed technique with 2/0 polypropylene Quill^TM^ SRS and *C* barbed technique with 2/0 PDO Quill^TM^ SRS): the average 2-mm gap formation force (*red bars*) and the breaking force (*blue bars*) are shown for each tendon repair technique

The two-sample Student *t*-test demonstrated a significant increase in mean load at 2-mm gap formation with barbed sutures, independently from suture material, when compared to a traditional Kessler suture. No statistically significant differences in mean load at 2-mm gap formation were registered between the two barbed groups. As regards load to failure, the two barbed groups demonstrated a significantly increased breaking force when compared to the Kessler group, and also the 4-strand technique with Quill™ SRS PDO suture demonstrated significantly better resistance to failure relative to the 4-strand repair with Quill™ SRS polypropylene suture (Tables 3, 4; Fig. 5). Note that the differences between pre-repair areas are not significant, except between the barbed group with Quill™ SRS PDO suture and the Kessler group where a significant difference emerges (Table 5). Nevertheless, this difference is irrelevant because we calculated the breaking force of the suture that is not affected by the area of the tendon. Indeed, in all tests the failure mode is due to the breakage of the suture or suture pull-out, but never due to tendon failure.

**Table 3:** Results of the two-sample Student *t*-test applied to 2-mm gap formation load

Student *t*-test/2-mm gap formation load			
	Group A	Group B	Group C
Group A	//	6.914 (*P* < 0.01)	6.893 (*P* < 0.01)
Group B		//	0.853 (*P* = 0.399)
Group C			//

**Table 4:** Results of the two-sample Student *t*-test applied to breaking force

Student *t*-test/breaking force			
	Group A	Group B	Group C
Group A	//	8.5 (*P* < 0.01)	11.759 (*P* < 0.01)
Group B		//	3.375 (*P* < 0.01)
Group C			//

**Table 5:** Results of the two-sample Student *t*-test applied to pre-repair area

Student *t*-test/pre-repair area			
	Group A	Group B	Group C
Group A	//	2.432 (*P* = 0.025)	3.981 (*P* < 0.01)
Group B		//	1.287 (*P* = 0.205)
Group C			//

## Discussion

Initially, the breaking force of tendon repair depends on the biomechanics of tendon sutures. Immobilized tendon sutures lose 50 % of their initial strength within the first week due to tenomalacia at the suture-tendon junction [8]. Early passive and especially active motion rehabilitation programs have been shown to prevent the initial weakening at the repair site by improving tendon nutrition, healing and remodeling [9, 10]. Therefore, it is absolutely essential that the tendon repair is sufficiently strong to tolerate the forces generated during early active motion, which are of 40–50 N as described by Amadioet al. [11].

The breaking force of the repair can be improved by increasing the number of strands crossing the repair site, the suture caliber and the number of knots; however, in this way the tendon cross-sectional area is enlarged, causing increased gliding resistance [12].

Consequently, the ideal suture technique must be strong enough to allow early active motion with minimal deformity of the cross-sectional area at the repair site.

All conventional tenorrhaphy techniques require knots, but knots are potential weak points in tendon sutures. If a knot lies within the tendon, it may reduce vascularization, tendon apposition and intrinsic healing, causing extrinsic neovascularization and adhesion formation. Furthermore, bulky knots enlarge the tendon cross-sectional area, increasing gliding resistance during active flexion and therefore the risk of gapping or suture failure.

The advantages of barbed sutures are to eliminate the need for a knot and to better distribute the load throughout the tendon repaired due to a greater number of points for barb-tendon interaction along the length of the suture. In this way, the bunching at the repair site is reduced and the breaking force improved.

Previous studies hypothesized that a knotless flexor tendon repair using bidirectional barbed suture has a similar breaking force to a traditional knotted technique but with a smaller change in the repair site cross-sectional area. This was proven by McClellan et al. [7] who compared, in a porcine model, two conventional techniques, the 2-strand Kessler and the 4-strand Savage, with a 4-strand barbed tenorrhaphy. By testing the 2-mm gap formation force and the load to failure, they demonstrated that Savage and barbed techniques have equivalent breaking force, both significantly greater than the Kessler method. As regards tendon deformity, the repair site cross-sectional area of tendon repaired with the knotless technique was significantly smaller than that of tendons repaired with Kessler and Savage techniques. Parikh et al. [5] compared, in cadaver flexor tendons, 3-strand and 6-strand barbed suture techniques to a knotted 4-strand cruciate technique, demonstrating that the 3-strand barbed suture achieved a breaking force comparable to that of 4-strand cruciate repair, but with significantly less repair site bunching. In the 6-strand barbed suture technique an increased breaking force and significantly less repair site bunching have been recorded, compared with 4-strand cruciate repair. When trying to critically analyze the literature, in each study one finds that the tendon repair technique, number of strands, suture material and suture diameter between control and experimental groups change, making it difficult to compare the results. Another disadvantage of these studies lies in the lack of cyclical testing that models in vivo situations more realistically than linear tests alone. Recently, Zeplin et al. [13] compared a knotted with a knotless tendon repair technique, applying linear and cyclical loads, without detecting any difference in breaking force between the two groups in both situations.

In our study, we wanted to test a new 4-strand repair technique using Quill™ SRS suture. The control group was represented by a modified Kessler technique. Although it is not appropriate to compare a 4-strand with a 2-strand tenorrhaphy, the purpose was to test a new technique using barbed suture against a well-studied, widely accepted standard in flexor tendon repair. To maximize the purchase of the barb of the suture on the tendon fibrils, the repair was designed to traverse the tendon several times perpendicular to the direction of the collagen fibers.

As regards the suture material, after testing barbed suture using Quill™ SRS polypropylene 2/0, it was decided to try an absorbable material, Quill™ SRS polydioxanone 2/0, since, according to data provided by the manufacturer, it should have a higher suture breaking force, i.e., 1.77 kgf (17.36 N) versus 0.96 kgf (9.42 N). Furthermore, we did not want to leave a non-absorbable barbed material in the repaired tendon indefinitely. Before performing the tendon repair, the breaking force of the suture materials was measured and a higher load to failure compared to the declaration of the manufacturer was recorded. This data could be related to a safety factor utilized by the manufacturer. According to Quill™ SRS’s manufacturer, the results of implantation studies in animals using PDO indicate that for sizes larger than 3/0, approximately 80 % of the original strength remains after 4 weeks of implantation. The absorption of PDO is declared be minimal until about 120 days and essentially complete within 180 days. However, additional in vivo studies are needed in order to understand better the biological behavior of this absorbable suture material, to determine whether it is absorbed prematurely or if it creates denser scarring.

In this study, a significant increase in mean load at 2-mm gap formation with barbed sutures was exhibited, independently of suture material, compared with a traditional Kessler suture. No statistically significant difference in mean load at 2-mm gap formation was registered between the two barbed groups. As regards load to failure, the two barbed groups demonstrated a significantly increased breaking force when compared to the Kessler group, and the 4-strand technique with Quill™ SRS PDO suture also had a significantly higher load to failure when compared with the 4-strand repair using Quill™ SRS polypropylene suture. In barbed tenorrhaphy using the Quill™ SRS suture, the breaking force of the repair exceeded the 40–50 N suggested by Amadio [11] as sufficient to initiate early active motion.

Concerning repair site profile, less bunching was recorded at the repair site with the barbed suture compared with the conventional modified Kessler technique. This result improves tendon gliding through the sheath, and avoids peripheral epitendinous suturing.

As regards the failure mode, it was observed that all barbed suture repairs failed by suture breakage, whereas unbarbed control repair failed in 40 % of cases by suture pull-out and in 60 % by suture breakage. This suggests that inadequate suture-tendon interaction was the limiting factor in achieving a high breaking force with the modified Kessler technique, whereas in barbed repair the native strength of the suture material, rather than slippage, was the weak point. By increasing the suture diameter or by applying barbs to materials with higher tensile strength, an improvement in repair site breaking force could be gained.

Despite the encouraging results of this study, it is acknowledged that a number of possible limitations and difficulties may exist with respect to the clinical application of this new barbed tenorrhaphy. Firstly, as this new technique was not performed in situ, it has not been possible to assess the ease of suturing in a clinical setting under the constraints of limited exposure, tendon retraction and tension, especially in zone II. Secondly, it has not been possible to assess in vivo factors such as tendon ischemia and healing after repair, edema, and adhesion formation of this new repair. Another critical aspect is that to maintain the integrity of the barbs, no direct handling of the suture is to be performed with fingers or instruments, so if there is a technical error during repair, the suture has to be cut and removed completely, since it is impossible to back up the suture to rethrow a stitch without damaging the barbs. Finally, our biomechanical testing used a linear load to failure, which may not reflect the physiologic conditions as well as cyclic loading models.

In conclusion, this study confirms the promising results achieved in previous studies concerning the use of barbed suture in flexor tendon repair. In our animal model, tenorrhaphy with Quill™ SRS suture guarantees a breaking force of repair that exceeds the 40–50 N suggested as sufficient to initiate early active motion, and a smoother profile of the repair site. Further in vivo testing is warranted to evaluate the clinical applicability of this new barbed suture tenorrhaphy, especially in zone II tendon flexor laceration, where a more aggressive rehabilitation plan is desired to reduce tendon adhesions and improve the functional outcome.

## Footnotes

1 The articles cited in the literature improperly use the term “tensile strength”, which is force per unit area, as they present the measured data in Newtons (the SI derived unit of force). Therefore, in this paper only the correct term of “breaking force” will be utilized.
